# An Integrated Process for the Xylitol and Ethanol Production from Oil Palm Empty Fruit Bunch (OPEFB) Using *Debaryomyces hansenii* and *Saccharomyces cerevisiae*

**DOI:** 10.3390/microorganisms10102036

**Published:** 2022-10-14

**Authors:** Efri Mardawati, Emilda Ayu Febrianti, Hana Nur Fitriana, Tri Yuliana, Norisca Aliza Putriana, Sri Suhartini, Kasbawati

**Affiliations:** 1 Faculty of Agro-Industrial Technology, Universitas Padjadjaran, Jatinangor 45363, Indonesia; 2 Research Collaboration Center for Biomass and Biorefinery between BRIN and Universitas Padjadjaran, Jatinangor 45363, Indonesia; 3 Faculty of Pharmacy, Universitas Padjadjaran, Jatinangor 45363, Indonesia; 4 Department of Agro-Industrial Technology, Faculty of Agricultural Technology, Universitas Brawijawa, Malang 65145, Indonesia; 5 Department of Mathematics, Universitas Hasanuddin, Makassar 90245, Indonesia

**Keywords:** OPEFB, lignocellulose, *Saccharomyces cerevisiae*, *Debaryomyces hansenii*, ethanol, xylitol, SSF

## Abstract

Oil palm empty fruit bunch (OPEFB) is the largest biomass waste from the palm oil industry. The OPEFB has a lignocellulose content of 34.77% cellulose, 22.55% hemicellulose, and 10.58% lignin. Therefore, this material’s hemicellulose and cellulose content have a high potential for xylitol and ethanol production, respectively. This study investigated the integrated microaerobic xylitol production by *Debaryomyces hansenii* and anaerobic ethanol semi simultaneous saccharification and fermentation (semi-SSF) by *Saccharomyces cerevisiae* using the same OPEFB material. A maximum xylitol concentration of 2.86 g/L was obtained with a yield of 0.297 g/g_xylose_. After 96 h of anaerobic fermentation, the maximum ethanol concentration was 6.48 g/L, corresponding to 71.38% of the theoretical ethanol yield. Significant morphological changes occurred in the OPEFB after hydrolysis and xylitol and ethanol fermentation were shown from SEM analysis.

## 1. Introduction

Indonesia is the largest palm oil producer and exporter in the world, having 16.3 million hectares of palm oil plantations and producing more than 35 million tons of palm oil in 2022 [1]. However, around 23% of every ton of oil palm fresh fruit bunch is converted into crude palm oil, and the remainder is turned into biomass waste, such as oil palm empty fruit bunches (OPEFB), fibers, and shells [2]. OPEFB waste has become an environmental problem because it must be burned or converted into compost for its final disposal [3]. OPEFB generation accounts for 23% of the fresh fruit bunches (FFB) production [4]. Only 10% of OPEFB is used as compost for soil amendment in oil palm plantations, while the remaining 90% of OPEFB is still unprocessed waste [5]. Good waste management is needed to manage this tremendous amount of OPEFB waste, so that it does not cause significant environmental problems in the future. One waste management strategy is utilizing the lignocellulosic content of OPEFB to produce a high-value-added product, such as xylitol and ethanol [6].

Several strategies were proposed and assessed to emphasize the technical and commercial opportunities for OPEFB conversion. These strategies were based on either mono-production of xylitol or bioethanol or combined production via process integration (co-production) [7]. The xylitol and ethanol co-production scenario is considered more profitable because this process increases the economics of low bioethanol production by adding another high-value product, namely xylitol [8]. In addition, from a biorefinery perspective, through this process, all hemicellulose and cellulose fractions can be appropriately utilized; thus, waste products can be minimized [9].

The lignocellulose content in OPEFB is reported to be 37.3–46.5% of cellulose, 25.3–33.8% of hemicellulose, and 20.4–32.5% of lignin [10,11]. Hemicellulose is a heteropolymer made primarily of pentose sugars (xylose and arabinose), hexose sugars (mannose, galactose, and glucose), uronic acids, and other sugars (rhamnose and fucose) [12]. Generally, hemicellulose hydrolysis can be easier and faster than cellulose because of its lower degree of polymerization and heterogeneous and amorphous structure [13]. The hemicellulose of OPEFB consists mainly of xylose, up to 19.6% of OPEFB, while arabinose, mannose, and galactose are detected at lower levels, i.e., at 1.9%, 1.4%, and 1.2%, respectively, indicating that OPEFB has the potential to be employed as a raw material for xylitol production [14]. Xylitol is a polyol sugar with five carbons, which is widely used in various pharmaceutical products as a sweetener [15]. Worldwide, xylitol is industrially manufactured by the catalytic hydrogenation of pure D-xylose solution under high temperature and pressure, which consumes much energy and cost production [16]. Therefore, the production of xylitol via microorganisms, which can be performed under mild conditions is expected to meet market demands with a more economic value [17]. Various microorganisms, such as bacteria, yeast, and fungi, were studied for their ability to convert xylose to xylitol [16,18,19,20]. Among these microorganisms, *Debaryomyces hansenii* is one of the best yeasts for generating xylitol in high concentrations [21,22].

Many studies reported the potential use of OPEFB as the feedstock of bioethanol production due to its high cellulose content [7,23,24,25]. *Saccharomyces cerevisiae* is the most commonly employed yeast for commercial ethanol production, although various yeasts, bacteria, and other microorganisms can produce ethanol [26,27]. Separated hydrolysis and fermentation (SHF) and saccharification and simultaneous fermentation (SSF) are two fermentation methods commonly used in bioethanol production [28]. However, the SSF method is often used to improve the efficiency of ethanol production by integrating the enzymatic hydrolysis of lignocellulosic material into ethanol fermentation in one process [29]. Saha et al. [30] reported that the ethanol yield from the lignocellulosic material of the SSF process was 16% higher than the yield obtained from the one conducted separately.

The co-production of xylitol and ethanol from many lignocellulosic materials, such as corncob [31], sisal fiber [32], sugarcane bagasse [27], and banana and water hyacinth leaves [33], have been investigated. However, there are few reports of the co-production of the metabolites using OPEFB as raw materials, even though the amount of OPEFB is abundant in agriculture waste, especially in Indonesia and the other largest palm oil producing countries. As a result, this study was carried out to investigate the OPEFB potential as xylitol and bioethanol feedstock by integrating the microaerobic xylitol production process by *Debaryomyces hansenii* and anaerobic ethanol production using the semi-SSF method by *Saccharomyces cerevisiae*. The maximum benefit of OPEFB can be obtained towards zero waste concept integration through the approach taken. The objective of this study was to evaluate whether this approach could result in high xylitol and ethanol yields and good growth in yeast cells associated with each process.

## 2. Materials and Methods

### 2.1. Tools and Materials

The oil palm empty fruit bunches (OPEFB) were obtained from Condong Co., Ltd., Garut, West Java, Indonesia. All parts of the OPEFB material were cleaned under running water and then oven dried at 60 °C for 24 h. The dry material was reduced to particle size using a disc mill and then sieved with 60 mesh or 0.25 mm size. After preparation, the material was kept at room temperature in sealed bags until use.

The xylanase and cellulase used in the enzymatic hydrolysis were the Cellic HTec2 enzyme and Cellic CTec2 enzyme (Novozymes, Copenhagen, Denmark) with the activity of 75 IU/mL and 130 FPU/mL, respectively. *Debaryomyces hansenii* ITBCCR85 was obtained from ITB culture collection (Institut Teknologi Bandung, Bandung, Indonesia), while purified *Saccharomyces cerevisiae* was obtained from commercial yeast for the bakery. Four ways streaking method was conducted several times to obtain a single cell colony.

All supplemental chemicals obtained from Sigma Aldrich (St. Louis, MO, USA) were aerobicof analytical grade and were utilized directly. An 18.2 MΩ·cm Milli-Q water (Millipore, St. Louis, MO, USA) was used to prepare all solutions.

### 2.2. Autohydrolysis OPEFB Pretreatment

Autohydrolysis OPEFB pretreatment was carried out in 250 mL Erlenmeyer glass flasks using 15 g of OPEFB in 100 mL of 0.05 M acetate buffer pH 5, autoclaving at 121 °C for 15 min. This autohydrolysis process was carried out on fresh OPEFB and the OPEFB solid residue from hemicellulose hydrolysis.

### 2.3. Hemicellulose Hydrolysis

The hydrolysis of pretreated OPEFB was carried out in a 250 mL glass flask with 50 IU/gram biomass of enzyme xylanase, then incubated in an incubator shaker at a temperature of 50 °C for 96 h. The hydrolysate was separated from OPEFB residue by using centrifugation at a speed of 6000 rpm for 20 min. The liquid hydrolysate was concentrated threefold by evaporation at 70 °C and used for xylitol fermentation.

### 2.4. Fermentation of OPEFB Hydrolyzate by D. hansenii

*Debaryomyces hansenii* seed was grown on growth medium, which has composition of 50 mL of xylose (20 g/L) and 50 mL of nutrient medium containing 9438 g/L (NH_4_)_2_SO_4_, 2.5 g/L KH_2_PO_4_, 0.05 g/L CaCl_2_·2H_2_O, 0.5 g/L M_g_SO_4_·7H_2_O, 0.5 g/L citric acid, 0.035 g/L FeSO_4_·7H_2_O, 0.0092 g/L MnSO_4_·7H_2_O, 0.011 g/L ZnSO_4_·7H_2_O, 0.001 g/L CuSO_4_·7H_2_O, 0.002 g/L CoCl_2_·6H_2_O, 0.0013 g/L Na_2_CoO_4_·2H_2_O, 0.002 g/L H_3_BO_3_, 0.0035 g/L KI, 0.0005 g/L Al_2_(SO_4_)_3_, 0.1 g/L Myo-inositol, 0.02 g/L Calcium-pantothenate, 0.005 g/L Thiamine hydrochloride, 0.005 g/L Pyridoxal hydrochloride, 0.005 g/L Nicotine acid, 0.001 g/L Aminobenzoic acid, 0.0001 g/L D-biotin. The seed was incubated in the shaker incubator at 30 °C for 48 h with a stirring speed of 150 rpm.

The xylitol fermentation started with aseptically mixing OPEFB concentrated hydrolysate, inoculum solution (10^6^ CFU/mL), and growth medium in a volume ratio of 2:2:3. In a 250 mL Erlenmeyer, the total working volume was 100 mL. Other fermentation conditions were microaerobic at pH 5 and 30 °C with a stirring speed of 150 rpm. The microaerobic condition was achieved by mixing nitrogen gas and air in the Erlenmeyer head space at a 1:5 volume ratio.

### 2.5. Semi-Simultaneous Saccharification and Fermentation (Semi-SSF) of Ethanol

The production of bioethanol from OPEFB residue using the simultaneous saccharification and fermentation (SSF) method was carried out in a 250 mL Erlenmeyer with a total working volume was 100 mL. The OPEFB residue and cellulase enzyme concentration was 10% (*w*/*v*) and 65 FPU/gram biomass, respectively, of the total citrate buffer solution of pH 5. The saccharification process was carried out in the incubator shaker at 50 °C for 96 h, with a stirring speed of 130 rpm.

After 96 h of the saccharification process, 14% (*v*/*v*) *S. cerevisiae* seed with a cell concentration of 10^6^ CFU/mL was added to start the fermentation process without filtering the hydrolyzate. The fermentation was carried out in the incubator shaker at 30 °C for 96 h, with a stirring speed of 100 rpm, and under anaerobic conditions. The anaerobic condition was achieved by purging the Erlenmeyer head space with 100% of nitrogen gas.

### 2.6. Analysis Method

Lignocellulose composition was analyzed by Van Soest [34] method where the analysis of the cellulose, hemicellulose, and lignin contents of the biomass was determined synchronously. The samples from hydrolysis and fermentation were examined using an HPLC system (Waters type 1515 pump; Autosampler type 2707, Milford, MA, USA) equipped with UV and refractive index (RI) detectors. An Aminex HPX-87H column and a RI detector were used to measure the concentrations of xylose, glucose, ethanol, and xylitol at 65 °C with 5 mM H_2_SO_4_ as the mobile phase and 0.6 mL/min as the flow rate. A calibration curve was used to convert the optical density at 650 nm, which was used to measure cell growth to dry cell weight (DCW). The evaluation of the OPEFB surface before and after a series of processes was observed using a scanning electron microscope (JEOL, JSM-6330F; Tokyo, Japan).

### 2.7. Data Interpretation

The hydrolysis yield measurement was calculated based on Equations (1)–(5) [9].

Hemicellulose hydrolysis efficiency (%):

(1) $$\frac{\mathrm{xylose}\;\left(t\right)}{xylose\;\left(theo\right)}\times 100\%$$

Maximum theoretical xylose from hydrolysis (xylose (theo)):

(2) $$\mathrm{Mass}\;\mathrm{of}\;\mathrm{lignocellulose}\;\left(g\right)\times \mathrm{hemicellulose}\;\mathrm{content}\;\mathrm{in}\;\mathrm{linocellulose}\;\times 0.88$$

The yield of biomass (Y_X/S_) (g/g)

(3) $$Y_{X/S}=-\frac{∆X}{∆S}=\frac{X-X_{o}}{S_{o}-S}$$

The product yield (Y_P/S_) (g/g)

(4) $$Y_{P/S}=-\frac{∆P}{∆S}=\frac{P-P_{o}}{S_{o}-S}$$

where xylose(t) is the xylose concentration (g/L) produced at t time, X is biomass concentration (g/L), S is substrate concentration (g/L), and P is product (xylitol or ethanol) concentration (g/L).

Xylose or glucose Utilization (%):

(5) $$\frac{∆S}{S_{0}}=\frac{S-S_{0}}{S_{0}}\times 100\%$$

Xylitol fermentation efficiency (%):

(6) $$\frac{\mathrm{xylitol}\;\left(t\right)}{xylitol\;\left(theo\right)}\times 100\%$$

The theoretical yield of xylitol (xylitol (theo)) is 0.9 mol of xylitol per mol of xylose utilized [35].

Ethanol fermentation efficiency (%):

(7) $$\frac{\mathrm{xthanol}\;\left(t\right)}{xthanol\;\left(theo\right)}\times 100\%$$

The theoretical yield of ethanol (ethanol (theo)) is 0.51 mol of ethanol per mol of glucose utilized [36].

### 2.8. Statistical Analysis

Student *t*-test was used to determine the statistical significance of all measurements. The data were presented in the form of mean ± standard deviation. Statistical significance was defined as a *p*-value of less than 0.05.

## 3. Results

### 3.1. Characterization of Used Oil Palm Fruit Empty Bunches (OPEFB)

The raw materials of OPEFB were characterized for the lignocellulose composition to determine the approximate amount of xylose and glucose obtained through the hydrolysis process. Table 1 shows the lignocellulose content of used OPEFB is 34.77% of cellulose, 22.55% of hemicellulose, and 10.58% of lignin. Therefore, this material’s hemicellulose and cellulose content have a high potential for xylitol and ethanol production, respectively.

### 3.2. OPEFB Hydrolysis using Xylanase

**Figure 1:**
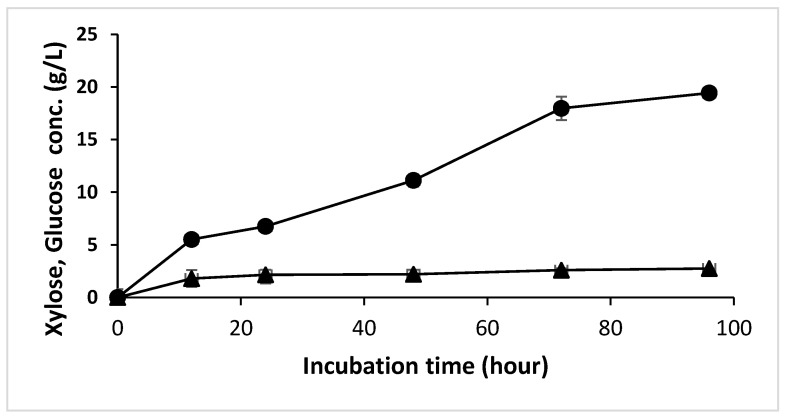
Xylose and glucose from OPEFB hydrolysis using xylanase. Xylose (solid circles), and glucose (solid triangles). Data presented are averages of triplicate experiments; error bars indicate the standard deviations.

Figure 1 shows an increase in xylose concentration over time, with the highest concentration being 19.42 g/L after 96 h of incubation. In comparison, the highest production rate of xylose was achieved in the first 12 h (0.42 g/L/h), suggesting that the xylanase enzyme was in the active condition and there is no inhibition from xylose yet. After 72 h, the activity of xylanase most likely decreased due to xylose inhibition [37,38]. Although the liberation of glucose, a byproduct of xylanase enzyme blend activity containing 11 FPU/mL of cellulase [39], increased significantly in the first 12 h, the increase in glucose concentration slowed down until the end of hydrolysis.

### 3.3. Growth and Xylitol Fermentation Profile of D. hansenii

**Figure 2:**
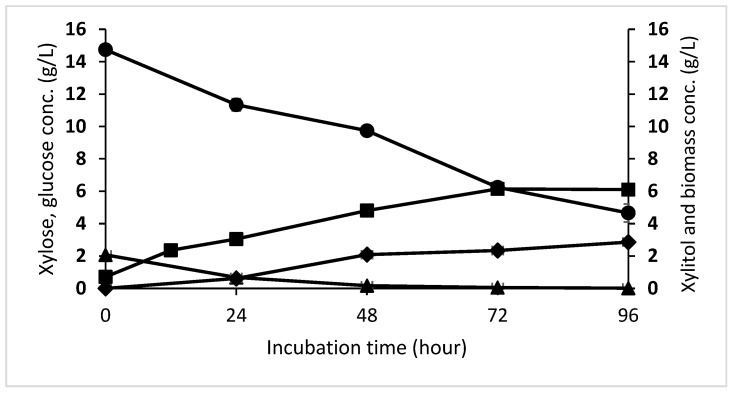
Xylitol fermentation profile. Xylose (solid circles), glucose (solid triangles), *D. hansenii* cell growth (solid squares), and xylitol (solid diamond). The data shown are the average of three replicate studies, and the error bars show the standard deviations.

*D. hansenii* grew well until 72 h by consuming 7.2 g/L of xylose and 2.01 g/L glucose and producing 6.1 g/L of dry cell weight and 2.34 g/L of xylitol (Figure 2). In the last 24 h of the fermentation process, cell growth was stagnant, but the cells still consumed 1.58 g/L xylose and produced 0.52 g/L xylitol. The xylitol fermentation process resulted in a yield of 0.297 g_xylitol_/g_xylose_ and a biomass yield of 0.46 g_cell_/g_xylose, glucose_ from the total xylose (14.74 g/L) and glucose (2.8 g/L) present in OPEFB hydrolysate.

### 3.4. Bioethanol Production by Semi-SSF Process using S. cerevisiae

**Figure 3:**
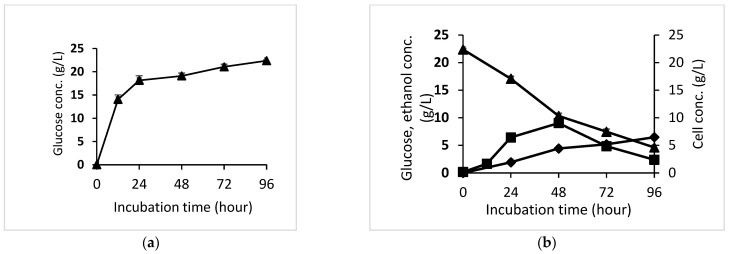
Glucose from OPEFB residue hydrolysis using cellulase (**a**); Ethanol fermentation profile (**b**), glucose (solid triangles), *S.cerevisiae* cell growth (solid squares), and ethanol (solid diamond). The data shown are the average of three replicate studies, and the error bars show the standard deviations.

The semi-SSF bioethanol process began with autohydrolysis of OPEFB solid residue. This stage aims to remove any remaining lignin in the OPEFB, increasing cellulose digestibility. The next stage was the enzymatic hydrolysis process for the first 96 h to ensure that the saccharification process occurred optimally. The highest glucose concentration obtained from cellulose hydrolysis was 22.37 g/L of glucose, as shown in Figure 3a. The enzyme activity appeared very fast in the first 12 h and gradually slowed until the hydrolysis process was completed.

Figure 3b depicts the exponential growth of *S.cerevisiae* until 48 h, followed by a decrease in cell growth until the end of the fermentation time. This cell growth pattern corresponds to the highest glucose consumption rate during the first 48 h of fermentation and then decreases throughout the rest of the fermentation time. In terms of the pattern of ethanol production, the accumulation factor in the reactor caused an increase in ethanol output over time. In summary, *S. cerevisiae* consumed 79.33% of the glucose in 96 h and produced ethanol up to 6.48 g/L, corresponding to a yield of 0.365 g_ethanol_/g_glucose_ and 71.38% of the theoretical ethanol yield.

### 3.5. Morphological Changes Occurred in OPEFB during the Reactions

**Figure 4:**
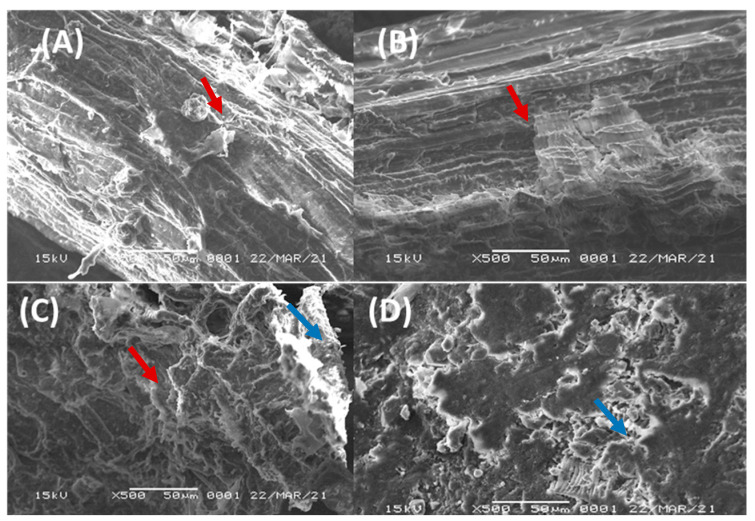
Scanning electron microscope images. Fresh OPEFB (**A**), after xylanase hydrolysis (**B**), after cellulase hydrolysis (**C**), after ethanol semi-SSF process (**D**). Lignin (red arrow), silica (blue arrow).

The morphological feature changes and surface characteristics of the OPEFB during each stage of xylitol and ethanol production were determined using SEM analysis. SEM images of OPEFB in fresh condition, after hemicellulose hydrolysis, cellulose hydrolysis, and ethanol fermentation, are shown in Figure 4. A comparison of SEM images reveals that these processes altered the structural makeup of biomass significantly. The main framework of plant cell walls, lignin, is still covering the surface of fresh OPEFB (Figure 4a). Because the lignin structure in OPEFB is very tight and robust, the enzymes will have a difficult time breaking down hemicellulose and cellulose into simple sugars [40].

The lignin layer is significantly reduced in fresh OPEFB, as shown in Figure 4b. The morphological structure of OPEFB after the second stage of hydrolysis with cellulase appeared more broken and open than the morphological structure after the first stage of hydrolysis with xylanase. Figure 4c depicts the structure of the broken lignin, as well as the surface of the silica-covered material. After ethanol fermentation, the morphological structure of OPEFB was found to be open and damaged. Figure 4d shows that the lignocellulosic component of the material, other than the silica layer, cannot be identified. During the semi-SSF process, the activity of *S. cerevisiae* and cellulase enzyme caused this damage.

## 4. Discussion

OPEFB is the enormous solid waste generated by the oil palm industry [41]. In the OPEFB scenario, lignocellulose xylitol is currently produced by fermenting xylose from biomass. The economics of this process scheme could be improved further by effectively utilizing the cellulose residue already present in the same lignocellulose feedstocks. Using separate cultures of *D. hansenii* and *S. cerevisiae*, this study demonstrated the potential co-production of xylitol and ethanol in the integrated process production of OPEFB feedstock.

The Van Soest method was used to analyze the lignocellulose components of the raw OPEFB from Java Island used in this study. The percentages of cellulose, hemicellulose, and lignin were 34.77 ± 1.20%, 22.55 ± 1.65%, and 10.58 ± 0.64%, respectively. However, the component percentages differed from those reported in a previous study using Sumatera Island OPEFB [38], which reported 43–43.47% cellulose, 22.93–23.67% hemicellulose, and 21.28–22.17% lignin. Variations could be caused by the variety of plant produce, growth conditions, and maturity level [42].

The hydrolysis process for xylitol production was preceded by autohydrolysis pretreatment of OPEFB raw materials. This method is highly effective in obtaining high xylose concentration [43], as well as delignification, which increases the porosity of OPEFB and enzyme access during enzymatic hydrolysis [44]. In the first enzymatic hydrolysis of OPEFB, xylanase was used due to its ability to hydrolyze xylan, which makes up the most content in hemicellulose structure [14]. After 96 h, the total xylose obtained was 19.42 g/L or 57% of theoretical xylose in the feedstock. As previously stated, this amount of xylose was also generated from autohydrolysis. In addition to xylose, a small amount of glucose (2.75 g/L) was produced as a byproduct of this hydrolysis process since the xylanase enzyme blend contained cellulase [39].

*Debaryomyces hansenii* was able to utilize xylose as the carbon source for cell growth and xylitol synthesis under microaerobic conditions since oxygen transfer coefficient and oxygen transfer rate significantly impact the xylose reductase activity in this microbe [45]. After 96 h of fermentation, 65% of the initial xylose was consumed, resulting in a xylitol yield of 0.297 g_xylitol_/g_xylose_ and a biomass yield of 0.56 g_cell_/g_xylose_. According to Kresnowati et al. [2], the high biomass yield obtained in this study suggested that OPEFB hydrolysate produced by enzymatic hydrolysis does not include any chemicals that inhibit xylitol fermentation by *D. hansenii* yeast. The xylitol and biomass yield in this study was even higher than those in the Kresnowati et al. [2] study using OPEFB hydrolysate, which resulted in 0.098 ± 0.029 g_xylitol_/g_xylose_ and 0.27 ± 0.09 g_cell_/g_xylose_, respectively.

Moreover, in this xylitol fermentation, glucose consumption was observed. The presence of glucose, in an optimal amount as a co-substrate, resulted in the rapid accumulation of active biomass and increased availability of redox co-factors [NAD(P)H], which can improve the production parameters of xylitol fermentation [46]. Mardawati et al. [47] reported that the optimum glucose to xylitol ratio was 25%, while a ratio greater than that value resulted in low xylitol yield and xylose utilization. In this study, the glucose to xylose ratio in OPEFB hydrolysate was 14%, which means that this amount of glucose contributed to increasing biomass and xylitol production.

The enzymatic hydrolysis process of the OPEFB solid residue resulted in 22.37 g/L of glucose. The semi-SSF process began when the yeast inoculum was added to start the fermentation process, leading to ethanol fermentation and hydrolysis of the remaining cellulose coinciding, thereby increasing ethanol production. Zhang et al. [48] reported that yeast grew significantly with an increase in initial substrate concentration when the glucose content was less than 160 g/L. The growth of yeast was observed since glucose is a crucial signal molecule and the most significant carbon source. For the yeast to obtain the optimal metabolism for growth and the best density based on substrate concentration, it can modify the expression and activity of its enzymes in response to their surroundings [49]. Within 96 h, *S. cerevisiae* consumed 79.33% of glucose and synthesized 6.48 g/L of ethanol, corresponding to a yield of 0.365 g_ethanol_/g_glucose_. However, the ethanol yield of this study was higher than that of Sukhang et al. [24], who obtained 0.281 g_ethanol_/g_glucose_ of yield using the SSF process by *Kluyveromyces marxianus* yeast. On the contrary, ethanol production rate and yeast growth decreased at the end of the fermentation time (72 h to 96 h), probably due to ethanol inhibition. Ethanol is a toxic metabolite that inhibits yeast cell growth and the amount of product synthesis [50,51]. Meanwhile, the morphological changes in the OPEFB structure during the hydrolysis and fermentation process revealed that the OPEFB was degraded in every single process stage.

Overall, the integration process of xylitol and ethanol production in this study is beneficial because it can save time producing these two metabolites. Semi-SSF ethanol production can be carried out simultaneously with xylitol fermentation. In addition, because the production of xylitol and ethanol is performed in different places, the process of separating and purifying the product can be readily completed. In addition, this process also used enzymes for the hydrolysis of hemicellulose and cellulose. Compared to chemical hydrolysis, enzymatic hydrolysis has several benefits, including reduced development of unfavorable byproducts, no requirement for corrosion-resistant processing material, being environmental friendly [52], working in mild conditions, reacting specifically, and the product can being easily integrated with the fermentation process [14,53]. In Sugiharto’s report, an enzyme-feeding strategy for fed-batch enzymatic hydrolysis of OPEFB was used to increase enzymatic digestibility and product concentration up to 26% and 12%, respectively, in less than 40 h [54]. Therefore, further investigation is needed to test the feasibility and practicability of using high solid loading (>15% *w*/*v*) by a fed-batch system for both OPEFB substrate and enzyme.

## 5. Conclusions

The integration of microaerobic xylitol production *by Debaryomyces hansenii* with anaerobic ethanol production by *Saccharomyces cerevisiae* using the same OPEFB material was proposed in this study. A maximum xylitol concentration of 2.86 g/L with a yield of 0.297 g/g xylose was obtained. In comparison, the maximum ethanol concentration was 6.48 g/L, corresponding to 71.38% of the theoretical ethanol yield. The benefits of the current method include processing solid pretreated material, which reduces solid waste of OPEFB, increasing the hemicellulose and cellulose potency in OPEFB to produce xylitol and ethanol and saving time because xylitol fermentation can be done concurrently with the ethanol semi-SSF process. These benefits can help make the lignocellulosic xylitol and ethanol industry more environmentally friendly and cost-effective, increasing the potential for industrial application.

## Figures and Tables

**Table 1:** Composition of lignocellulosic content of OPEFB.

Composition	Composition (%)
NDF ^1^	68.49 ± 1.53
Hemicellulose	22.55 ± 1.65
ADF ^2^	45.94 ± 0.83
Cellulose	34.77 ± 1.20
Lignin	10.58 ± 0.64
Silica	0.6 ± 0.14

^1^ Neutral detergent fiber; ^2^ Acid detergent fiber.

## Data Availability

All outcome data are available as representative images in the main text. The raw datasets generated analyzed during the current study are available from the corresponding author on reasonable request.
